# Methicillin-resistant *Staphylococcus aureus* isolates derived from humans and animals in Yogyakarta, Indonesia

**DOI:** 10.14202/vetworld.2023.239-245

**Published:** 2023-01-31

**Authors:** Mulya Fitranda, Siti Isrina Oktavia Salasia, Osman Sianipar, Dion Adiriesta Dewananda, Adika Zhulhi Arjana, Fatkhanuddin Aziz, Madarina Wasissa, Fajar Budi Lestari, Christin Marganingsih Santosa

**Affiliations:** 1 Department of Clinical Pathology, Faculty of Veterinary Medicine, Universitas Gadjah Mada, Yogyakarta, Indonesia; 2 Department of Clinical Pathology and Laboratory Medicine, Faculty of Medicine, Public Health and Nursing, Universitas Gadjah Mada, Yogyakarta, Indonesia; 3 Department of Bioresources Technology and Veterinary, Vocational College, Universitas Gadjah Mada, Yogyakarta, Indonesia

**Keywords:** antimicrobial resistance, methicillin-resistant *Staphylococcus aureus*, multi-drug resistance, *Staphylococcus aureus*

## Abstract

**Background and Aim:**

The emergence of methicillin-resistant *Staphylococcus aureus* (MRSA) as a highly pathogenic strain in veterinary and human medicine is a growing global problem. This study aimed to evaluate MRSA isolates of human and animal origin against various antibiotics in Yogyakarta, Indonesia.

**Materials and Methods:**

The susceptibility test was carried out by the disk diffusion method using Mueller-Hinton agar against nine antibiotic disks. Methicillin-resistant *S. aureus* strains were genetically confirmed through *mec*A gene detection encoding for methicillin resistance by polymerase chain reaction.

**Results:**

All 240 *S. aureus* strains isolated from animals and humans were resistant to penicillin G (P) (100% and 99%, respectively), followed by ampicillin (AMP), amoxicillin (AML), oxacillin (OX), erythromycin (E), clindamycin (DA), tetracycline (TE), gentamicin (GEN), and ciprofloxacin (CIP). Eighty-three MRSA strains were resistant to OX (100%), P (100%), AMP (99.27%), AML (95.52%), E (87.77%), TE (71.33%), DA (63.24%), GEN (38.81%), and CIP (26.87%).

**Conclusion:**

The antimicrobial resistance pattern of *S. aureus* human isolates was similar to their animal counterpart, with 77.20% of MRSA strains classified as multidrug-resistant (MDR) bacteria. These findings indicate an increase in MDR *S. aureus* strains of animal origin in Yogyakarta, thus raising public health concerns about MRSA zoonotic spread.

## Introduction

*Staphylococcus aureus*, which has evolved into methicillin-resistant *S. aureus* (MRSA), is a bacterium resistant to various antibiotics, also known as a superbug [1, 2]. Methicillin-resistant *S. aureus* is recognized as a major problem in hospitals worldwide [3]*. Staphylococcus aureus* resistance can interfere with the wound-healing process, leading to a prolonged wound-healing process, and increased mortality and morbidity [4, 5]. Estimated costs due to multidrug-resistant (MDR) bacterial infections might result in extra healthcare costs and productivity losses. The prevalence of MRSA infections is increasing worldwide; from 94,000 infection cases in America, the morbidity rate of MRSA infections has reached 18,650 cases. The prevalence of MRSA infections in Asia reaches 70%, whereas the prevalence of MRSA infections in Java, and Bali, Indonesia, reaches 3.1% [6]. Asia is a region with the highest prevalence rates of healthcare- and community-associated MRSA worldwide. Most hospitals in Asia are endemic for MDR *S. aureus*, with proportions estimated to be ~28% (in Hong Kong and Indonesia) to >70% (in Korea) among all clinical *S. aureus* isolates in the early 2010s [7, 8]. In western countries, novel MRSA from farm animals is considered a public health threat. The prevalence of MRSA in bovine milk ranged from 1.1% in Japan [9] to 52.2% in Egypt [10]. There is a high prevalence of *S. aureus* and MRSA isolated from milk of dairy water buffaloes with mastitis in the Philippines. Most MRSA isolates are still susceptible to common antibiotics. Nevertheless, ~37.5% of the isolates were considered MDR [11]. The emergence of MRSA in livestock has been implicated in the use of antimicrobials as growth promoters and for preventive and therapeutic measures [12–14].

There is a great concern for mastitis treatment caused by MRSA because MRSA is resistant to β-lactams and exhibits MDR patterns to other commonly used antibiotics [15, 16]. Moreover, there is an increasing concern about the public health risk of MRSA from livestock because resistance genes can spread from food animals to humans by direct contact or through food chain circulation [14, 17]. Antibiotics used in agriculture are often the same or similar to antibiotic compounds used clinically [18]. If not controlled properly, this overreliance on similar compounds in both fields can increase the risk of antimicrobial resistance in veterinary and human medicine. High MRSA levels in hospitals and the community as well as in livestock have led to an increased economic burden on the healthcare sector and the use of anti-MRSA agents, further promoting the development of resistant strains. Effective infection control strategies and choosing the appropriate antimicrobial agents for each cultured isolate remain the best methods to prevent further MRSA transmission and alleviate the burden of associated diseases.

The evaluation of *S. aureus* isolates of animal and human origin needs to be carried out continuously to determine the resistance developments of these bacteria so that control strategies can be pursued. This study aimed to evaluate MRSA isolates of human and animal origin against various antibiotics in Yogyakarta, Indonesia.

## Materials and Methods

### Ethical approval

Ethical permission (consent form) was obtained from the main management of the laboratory for providing human isolates and from the farmers for collecting milk samples.

### Study period and location

This study was conducted from February 2021 to June 2022 at the Clinical Pathology Laboratory, Faculty of Veterinary Medicine, Universitas Gadjah Mada.

### Bacterial isolates

*Staphylococcus aureus* strains (240) used in this study were retrieved from the Department of Clinical Pathology and Laboratory Medicine, Faculty of Medicine, Public Health and Nursing (174 human isolates) and the Department of Clinical Pathology, Faculty of Veterinary Medicine (66 animal isolates), Universitas Gadjah Mada, Yogyakarta, Indonesia. Clinical human and animal samples were streaked onto a blood agar base containing 5% defibrinated fresh sheep blood and incubated at 37°C for 18–24 h. Colonies consistent with *S. aureus* were streaked to single colonies and identified according to their colony morphology and biochemical and microbiological test results. All samples were also identified based on Gram staining, fermentation on mannitol salt agar (MSA), and catalase and coagulase tests. The catalase test was performed by placing a drop of hydrogen peroxide (H_2_O_2_) on a microscope slide. A small amount of bacterial isolate was added to H_2_O_2_, and oxygen bubbles were observed as catalase-positive. The coagulase test was performed by cultivating the bacteria in the tube coagulase test using rabbit plasma. Coagulation was observed at 6 and 24 h. *Staphylococcus aureus* (ATCC 25923) was used as a quality-control organism.

### Antimicrobial susceptibility test

All *S. aureus* isolates were inoculated into the nutrient broth (NB; Oxoid UK) and incubated at 37°C for 18–24 h. The cultures were diluted with fresh NB to give a turbidity equivalent to 0.05 McFarland. Susceptibility tests were performed by the disk diffusion method of the Kirby–Bauer test, as described by the Clinical and Laboratory Standards Institute (CLSI) [19], using Mueller-Hinton agar (Difco, USA) supplemented with 20 g/L NaCl. The antibiotics disks (Oxoid, UK) used were gentamicin (GEN) 10 μg, ampicillin (AMP) 10 μg, oxacillin 5 (OX) μg, tetracycline (TE) 30 μg, clindamycin (DA) 10 μg, penicillin G (P) 10 μg, erythromycin (E) 15 μg, and amoxicillin (AML) 25 μg. Inhibition zones were measured after 18 and 24 h incubation at 35°C. OX was used as an indicator of methicillin susceptibility disks. An inhibition zone diameter of ≤14 mm was reported as methicillin-resistant, 15–17 mm as intermediate, and ≥18 mm as methicillin-sensitive. The isolates were reported as sensitive, intermediate, and resistant based on CLSI guidelines.

### DNA isolation and purification

A QIAmp DNA mini kit (Qiagen, Germany) was used to purify DNA from *S. aureus* according to the manufacturer’s protocol. The bacterial strains were cultivated on blood agar base (Oxoid, Germany) containing 5% defibrinated sheep blood for 24 h at 37°C. Five to 10 *S. aureus* colonies were suspended with 180 μL TE buffer (10 mM Tris-HCl and 1 mM ethylenediaminetetraacetic acid [EDTA] [pH 8]) containing 5 μL lysostaphin (1.8 U/μL; Sigma, USA) in 2 mL microfuge tubes. The suspension was incubated for 1 h at 37°C, and 25 μL proteinase K (14.8 mg/mL; Sigma) and 200 μL AL buffer (containing AL1 and AL2 reagents; Qiagen) were then added. The suspensions were incubated for 30 min at 56°C and then for 10 min at 95°C before being spun at 6000× *g* for a few seconds. A total of 420 μL ethanol was added to each sample and placed in a spin QIAmp column. After centrifugation at 6000× *g* for 1 min, the spin columns were placed in a clean collection tube, and the sample was washed twice with 500 μL AW buffer (Qiagen). After the second wash and centrifugation at 6000× *g* for 3 min, the QIAmp spin columns were placed in a clean 2 mL microfuge tube, and DNA was eluted twice with 200 and 100 μL AE buffer (Qiagen). DNA was stored at −20°C.

### Molecular identification

Molecular identification was made according to the amplification of the *23S rRNA* and *nuc* genes with polymerase chain reaction (PCR) with the program and primer design described previously in Table-1 [20]. The reaction mixture (25 μL) contained 1 μL primer 1 (20 pmol), 1 μL primer 2 (20 pmol; IDT, USA), 12.5 μL PCR mix containing Taq DNA polymerase, MgCl_2_ and deoxynucleotide triphosphates (MyTaq Red Mix, Bioline, UK), 2 μL DNA template, and 8.5 μL distilled water. DNA of the isolates was prepared with the QIAmp DNA mini kit (Qiagen) as described by the manufacturer. Gene amplification was carried out with a thermal cycler (Benchmark, UK). The PCR products were separated by gel electrophoresis in a 1.5% (w/v) agarose gel (Invitrogen, USA) in 1 × TBE buffer (containing Tris base, boric acid, and EDTA). A 100 bp DNA ladder (Invitrogen) was used as a size marker. The resulting bands were visualized using Redsafe (Intron, Korea) staining under ultraviolet transillumination.

**Table-1:** Oligonucleotide primers and PCR programs used for amplifying the genes encoding *23S rRNA* [20], *nuc* [20]*,* and *mec*A [21] genes.

Target gene	Sequence (5´ -3´)	PCR program
*23S rRNA*	ACG GAG TTA CAA AGG ACG AC AGC TCA GCC TTA ACG AGT AC	35 cycles of 95°C for 15 s, 64°C for 30 s, and 72°C for 10 s
*nuc*	GCG ATT GAT GGT GAT ACG GTT ACG CAA GCC TTG ACG AAC TAA AGC	37 cycles of 94°C for 60 s, 55°C for 30 s, and 72°C for 5 s
*mecA*	AAA ATC GAT GGT AAA GGT TGG AGT TCT GCA GTA CCG GAT TTG	35 cycles of 95°C for 30 s, 55°C for 30 s, and 72°C for 10 s

PCR=Polymerase chain reaction

### Detection of the *mec*A gene encoding MRSA

Methicillin-resistant *S. aureus* strains were considered from MDR *S. aureus* against five or more antibiotics, including OX (50% MDR) from the nine antibiotics used in this study. The selected strains were confirmed by detecting the *mec*A gene encoding for methicillin resistance by PCR, as described previously by Widianingrum *et al*. [21]. The primers used to detect the *mec*A gene are listed in Table-1.

## Results

### *Staphylococcus aureus* strains

**Figure-1:**
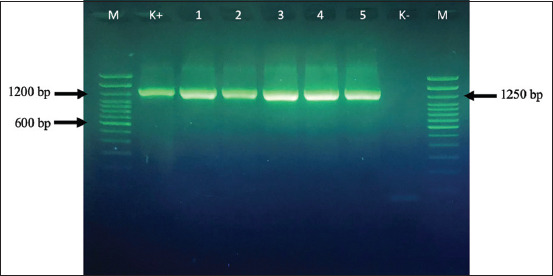
Amplicons of the gene encoding 23SrRNA of selected *Staphylococcus aureus* strains with a molecular size of 1250 bp. Lane K+: *S. aureus* control strain, Lane 1–5: selected *S. aureus*, Lane K-: negative isolate. Lane M: 100 bp molecular-size DNA ladder.

**Figure-2:**
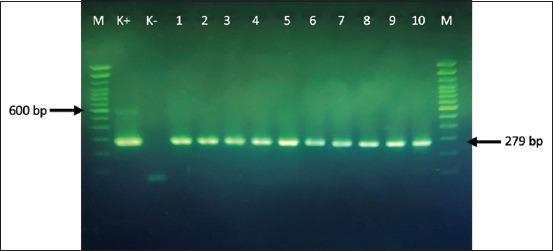
Amplicons of the nuclease (*nuc*) gene of selected *Staphylococcus aureus* strains, with a molecular size of 279 bp. Lane K+: *S. aureus* control positive strain, Lane 2: A *negative* isolate, Lane 3-10: *S. aureus* selected strains. Lane M: 100 bp molecular-size DNA ladder.

Based on the results of cultural and biochemical properties, along with amplification of the *nuc* and *23S rRNA* genes specific to *S. aureus*, all 240 human and animal isolates examined in this study were identified as *S. aureus*. All 240 cultures investigated were Gram-positive and positive for catalase, coagulase, and fermented mannitol in MSA. The identification of the isolates was then confirmed by PCR amplification of the species-specific gene encoding *23S rRNA* and the thermonuclease *nuc* gene. The amplicons of these genes showed a uniform size of ~1250 and 279 bp, respectively (Figures-1 and 2).

### Antimicrobial resistance

**Figure-3:**
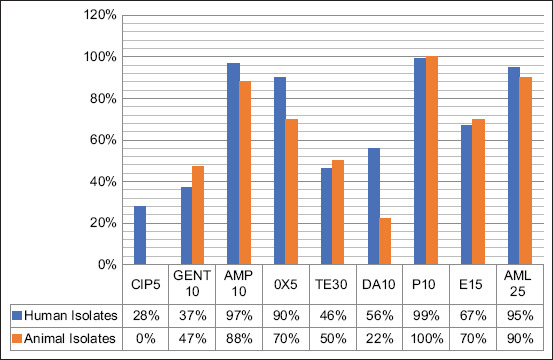
Resistance of *Staphylococcus aureus* isolated from humans and animals against several antibiotics. CIP5=Ciprofloxacin (5 µg), GENT10=Gentamicin (10 µg), AMP10=Ampicillin (10 µg), OX5=Oxacillin (5 µg), TE30=Tetracycline (30 µg), DA10=Clindamycin (10 µg), P10=Penicillin G (10 µg), E15=Erythromycin (15 µg), AML25=Amoxycillin (25 µg).

**Figure-4:**
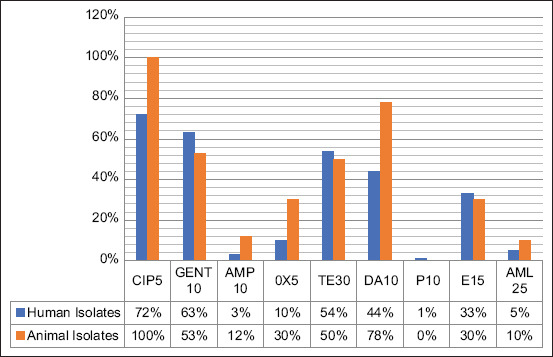
Susceptibility of *Staphylococcus aureus* isolated from humans and animals against several antibiotics. CIP5=Ciprofloxacin (5 µg), GENT10=Gentamicin (10 µg), AMP10=Ampicillin (10 µg), OX5=Oxacillin (5 µg), TE30=Tetracycline (30 µg), DA10=Clindamycin (10 µg), P10=Penicillin G (10 µg), E15=Erythromycin (15 µg), AML25=Amoxycillin (25 µg).

*Staphylococcus aureus* isolates were subjected to an antibiotic susceptibility test against nine antimicrobial agents. Antibiotics commonly used in human and veterinary medical cases in relevance were also considered. The resistance percentage of *S. aureus* against nine antibiotics is summarized in Table-2. The results of antimicrobial susceptibility are shown in Figure-3. *Staphylococcus aureus* strains were resistant to P (99% and 100%), followed by AMP (97% and 88%), AML (95% and 90%), OX (90% and 70%), E (67% and 70%), DA (56% and 22%), TE (46% and 50%), GEN (37% and 47%), and ciprofloxacin (CIP) (28% and 0%) for human and animal isolates, respectively (Figure-3 and Table-2). *Staphylococcus aureus* isolated from humans and animals were still susceptible to CIP (72% and 100%), GEN (63% and 53%), TE (54% and 50%), and DA (44% and 78%), respectively (Figure-4 and Table-2).

**Table-2:** Antibiotic susceptibility of *Staphylococcus aureus* isolated from humans and animals (bovine and goat) in Indonesia.

Antibiotic	Resistant (%)		Sensitive (%)	
	
	Human (n = 174)	Animals (n = 66)	Human (n = 174)	Animals (n = 66)
CIP (5 μg)	48/174 (28%)	0/20 (0%)	125/174 (72%)	20/20 (100%)
GEN (10 μg)	64/173 (37%)	23/49 (47%)	109/173 (63%)	26/49 (53%)
AMP (10 μg)	169/173 (97%)	58/66 (88%)	5/173 (3%)	8/66 (12%)
OX (5 μg)	156/173 (90%)	46/66 (70%)	18/173 (10%)	20/66 (30%)
TE (30 μg)	80/174 (46%)	33/66 (50%)	94/174 (54%)	33/66 (50%)
DA (10 μg)	98/174 (56%)	8/37 (22%)	76/174 (44%)	29/37 (78%)
P (10 μg)	173/174 (99%)	37/37 (100%)	1/174 (1%)	0/37 (0%)
E (15 μg)	116/174 (67%)	46/66 (70%)	58/174 (33%)	20/66 (30%)
AML (25 μg)	165/174 (95%)	18/20 (90%)	8/174 (5%)	2/20 (10%)

CIP=Ciprofloxacin, GEN=Gentamicin, AMP=Ampicillin, OX=Oxacillin, TE=Tetracycline, DA=Clindamycin, P=Penicillin G, E=Erythromycin, AML=Amoxycillin

### Multidrug-resistant in MRSA isolates

**Figure-5:**
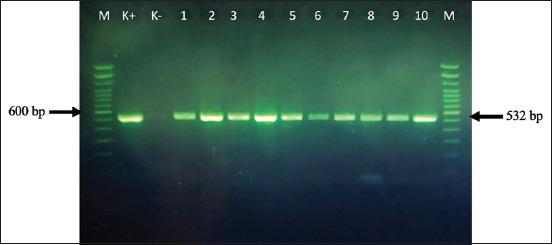
Polymerase chain reaction (PCR) product of *Staphylococcus aureus-*specific *mec*A gene 532 bp. A positive control (Lane K+), negative control (Lane K-no band), Lane 1–10: PCR product of *mec*A gene 532 bp for the selected isolates was applied, which showed clear bands confirmed that all the isolates were MRSA, Lane M: 100 bp molecular-size DNA ladder.

Methicillin-resistant *S. aureus* strains were considered MDR *S. aureus* when resistant for more than five antibiotics, including OX (50% MDR) from the nine antibiotics used in this study. Eighty-three MRSA-selected strains with MDR were identified by detecting the *mec*A gene encoding for methicillin resistance by PCR. The resistance pattern of MRSA strains is shown in Table-3. Figure-5 shows a positive control (MRSA), a PCR product of the *S. aureus*-specific *mecA* gene 532 bp (lane K+). A negative control PCR product applied on lane K- showed no band on the figure. The PCR product of the *mecA* gene 532 bp for the selected isolates was applied on lanes 1–10, which showed clear bands confirming that all isolates were MRSA. Methicillin-resistant *S. aureus* strains were resistant to OX (100%), P (100%), AMP (99.27%), AML (95.52%), E (87.77%), TE (71.33%), DA (63.24%), GEN (38.81%), and CIP (26.87%). The mean of MRSA strains resistant to nine antibiotics was ~77.20% (Table-3).

**Table-3:** Antibiotic resistance of the MRSA-selected isolates from humans and animals (n = 83).

Source of isolates	*mecA* gene 532 bp	Resistance to antibiotics									% MDR

		CIP5	GENT10	AMP10	OX5	TE30	DA10	P10	E15	AML25	
Human (n = 68)	+	18/67 (26.87%)	26/67 (38.81%)	67/68 (98.53%)	68/68 (100%)	29/68 (42.65%)	43/68 (63.24%)	68/68 (100%)	50/68 (73.53%)	64/67 (95.52%)	71.02%
Animal (n = 15)	+	0	0/15 (0%)	15/15 (100%)	15/15 (100%)	15/15 (100%)	0	15/15 (100%)	15/15 (100%)	0	83.33%
Total isolates (n = 83)	100%	26.87%	38.81%	99.27%	100%	71.33%	63.24%	100%	87.77%	95.52%	77.20%*

CIP5=Ciprofloxacin (5 µg), GENT10=Gentamicin (10 µg), AMP10=Ampicillin (10 µg), OX5=Oxacillin (5 µg), TE30=Tetracycline (30 µg), DA10=Clindamycin (10 µg), P10=Penicillin G (10 µg), E15=Erythromycin (15 µg), AML25=Amoxycillin (25 µg). R=Resistant, S=Sensitive, 0=Not determined, MDR=Multi-drug resistance,

* Mean of MRSA strains resistant to 9 antibiotics=77.20%. MRSA=Methicillin-resistant *Staphylococcus aureus*, MDR=Multidrug-resistant

## Discussion

According to biochemical test results, along with the amplification of the *nuc* and *23S rRNA* genes specific to *S. aureus*, all 240 human and animal isolates examined in the study were identified as *S. aureus*. The identification of the isolates was confirmed through PCR amplification of species-specific genes encoding *23S rRNA* (1250 bp) and the thermonuclease *nuc* gene (279 bp). The presence of these two genes can be used as a specific marker for *S. aureus* species [22, 23].

*Staphylococcus aureus* isolated from human and animal cases in this study were multi-resistant to various antibiotics, such as P, AMP, AML, OX, and E, with a resistance range of 70%–100%, indicating a high prevalence of MDR bacteria occurring in Yogyakarta (Table-2 and Figure-3). It is also particularly interesting that the antimicrobial resistance pattern of *S. aureus* human isolates is similar to animal isolates. This finding indicates an increase in MDR strains among *S. aureus* of animal origin in Yogyakarta, especially compared to a previous similar study in Indonesia, indicating that the antibiotic resistance of *S. aureus* isolates from humans (80%) was higher than bovine (76.92%) and goat (41.67%) [21]. *Staphylococcus aureus* isolates from animals in Indonesia were resistant to AMP, GEN, TE, and OX. These drugs are commonly used in veterinary medicine in Indonesia [21]. *Staphylococcus aureus* is also considered the primary cause of mastitis in cattle, water buffaloes, and goats in Asia [11, 18, 21]. In Egypt [24], 100% of *S. aureus* isolates were resistant to AMP and OX from 77 isolates of cow and buffalo milk suspected of MRSA and 84.8% of *S. aureus* were resistant to AMP and 74.1% to OX from 112 isolates of cow and buffalo milk.

*Staphylococcus aureus* is resistant to β-lactams, such as AMP, P, TE, and OX [16, 24]. Widianingrum *et al*. [21] reported an increase in the resistance of *S. aureus* to AMP, GEN, TE, and OX, indicating that the use of antibiotics commonly used in humans, now also widely used in animals, contributes to the current similar resistance phenomenon. The resistance pattern that originally occurred only in the human medical field now also occurs in animals. Improper hygiene and poor farm management practices contributed to the presence of *S. aureus* in the milk [8, 24, 25]. The hand-milked technique of dairy animals can also be a possible mode of transmission leading to the high prevalence of *S. aureus* infections [11].

With the increasing resistance of *S. aureus* strains to various antibiotics, it is necessary to constantly evaluate the use of antibiotics in humans and animals. Prescribing antibiotics is inappropriate regarding which compounds to use, the dosage, and the duration of administration will cause severe effects, particularly bacterial resistance. Antimicrobial resistance can be challenging to control and develop rapidly, thus further contributing to possible bacterial infection outbreaks in a population. A bacterial sensitivity test using various antibiotics determines the right antibiotic before prescribing a treatment plan. This study indicated that *S. aureus* isolates collected mainly from Yogyakarta are still susceptible to CIP for humans and animals (72% and 100%), GEN for humans (63%), and DA for animals (78%; Table-2 and Figure-4).

*Staphylococcus aureus* strains were once almost uniformly susceptible to β-lactams resistant to semisynthetic P (e.g., methicillin and OX), the class of antibiotics most commonly used for skin infections. This particular strain is widely known as MRSA, which implies that the bacteria already have cross-resistance to all β-lactams, including all P and cephalosporin [16]. The MRSA strains in this study were confirmed by detecting the *mecA* gene encoding for methicillin resistance with PCR. This finding was further supported by the disk diffusion test result in which all isolates tested were resistant to most of the nine antibiotics used in the study. Eighty-three MRSA human and animal strains in this study were resistant to OX (100%), P (100%), AMP (99.27%), AML (95.52%), E (87.77%), TE (71.33%), DA (63.24%), GEN (38.81%), and CIP (26.87%). The antimicrobial resistance pattern of *S. aureus* animal isolates is similar to human isolates. From these data, the mean of MRSA strains of human and animal isolates resistant to nine antibiotics is ~77.20% (Table-3). Widianingrum *et al*. [21] reported that the average of the prevalent MRSA strains of human and animal origin in Indonesia is 35.7%. This study confirmed that the prevalence of MRSA increased compared to previous studies [6, 21]. For many years, MRSA has been considered only a human pathogen until reports of MRSA mastitis (udder infection) in dairy cattle appeared in 1972 [26]. It has become increasingly important in veterinary medicine, with MRSA infections commonly reported in companion and farm animals [21, 27].

This finding indicates an increased prevalence of MDR MRSA among *S. aureus* of animal origin and the spread of MRSA among hospital-acquired and livestock-associated infections in Yogyakarta. In 2006, the prevalence of MRSA infections in Asia reached 70%, whereas it reached 3.1% in Java and Bali [6]. In early 2010, most hospitals in Asia were endemic for MDR MRSA, with reported proportions estimated from 28% (in Hong Kong and Indonesia) to >70% (in Korea) among all clinical *S. aureus* isolates [8].

This study has limitations, as this study could not determine whether original infections in human medical cases were acquired from zoonotic disease or as a nosocomial infection from a hospital. This study provided an opportunity to find a correlation between MRSA in dairy cattle and goats and the possibility of infection in workers close to dairy animals. The correlation of MRSA infections between livestock and workers will significantly contribute to the spreading pattern of MRSA infections, as reported previously in a study of MRSA prevalence among workers and dairy cattle in the Italian province of Ragusa [28]. Methicillin-resistant *S. aureus* infection cases in Indonesia have not been widely studied in humans and animals. The closeness between humans and animals, livestock, and products of animal origin that are consumed allows the transmission of *S. aureus* or MRSA infections [17, 20].

As reviewed by Chen and Huang [8], MDR *S. aureus* remains an important medical organism and is associated with a considerable disease burden in Asia. The high transmissibility of *S. aureus* strains in crowded living conditions in Asia constitutes a substantial public health threat, especially in resource-poor countries, where diagnostic facilities are primarily lacking, and appropriate therapy is frequently unaffordable. Implementation of surveillance systems at the international level is urgently needed to gain insights into the current epidemiology of *S. aureus* in resource-limited Asian countries.

This study on MRSA from human and animal isolates, including MRSA strains in Yogyakarta, described the development of the resistance of these bacteria, which could be used to pursue antimicrobial resistance control strategies.

## Conclusion

*Staphylococcus aureus* isolated from humans and animals used in this study were MDR against nine antibiotics in the range of 71.02%–83.33%, indicating the high prevalence of MDR currently occurring in Yogyakarta. It is also particularly interesting that the antimicrobial resistance pattern of *S. aureus* human isolates is similar to their animal counterparts. These findings indicated an increased MRSA prevalence among *S. aureus* of animal origin in Yogyakarta. The MRSA strains observed in this study revealed ~77.02% MDR, further confirming the spread of MRSA in animal and human medicine in Yogyakarta, Indonesia.

## Authors’ Contributions

MF, SIOS, OS, DAD, AZA, FA, MW, FBL, and CMS: Contributed to the conception of the study, designed, conducted the experiments, and analyzed the data. MF, OS, DAD, AZA, and MW: Contributed to sample preparation. SIOS, FA, and FBL: Drafted the manuscript. All authors have read and approved the final manuscript.
